# Adaptation and Implementation of a Shared Decision-Making Tool From One Health Context to Another: Partnership Approach Using Mixed Methods

**DOI:** 10.2196/42551

**Published:** 2023-07-05

**Authors:** Sophie Turnbull, Nicola E Walsh, Andrew J Moore

**Affiliations:** 1 Population Health Sciences University of Bristol Bristol United Kingdom; 2 Centre for Health and Clinical Research University of the West of England Bristol United Kingdom; 3 Musculoskeletal Research Unit School of Clinical Sciences University of Bristol Bristol United Kingdom

**Keywords:** shared decision-making, implementation, theoretical domains framework, qualitative, osteoarthritis, digital, mixed methods study, decision-making, disability, treatment, tool, effectiveness, acceptability, users, design

## Abstract

**Background:**

Osteoarthritis is a leading cause of pain and disability. Knee osteoarthritis accounts for nearly four-fifths of the burden of osteoarthritis internationally, and 10% of adults in the United Kingdom have the condition. Shared decision-making (SDM) supports patients to make more informed choices about treatment and care while reducing inequities in access to treatment. We evaluated the experience of a team adapting an SDM tool for knee osteoarthritis and the tool’s implementation potential within a local clinical commissioning group (CCG) area in southwest England. The tool aims to prepare patients and clinicians for SDM by providing evidence-based information about treatment options relevant to disease stage.

**Objective:**

This study aimed to explore the experiences of a team adapting an SDM tool from one health context to another and the implementation potential of the tool in the local CCG area.

**Methods:**

A partnership approach using mixed methods was used to respond to recruitment challenges and ensure that study aims could be addressed within time restrictions. A web-based survey was used to obtain clinicians’ feedback on experiences of using the SDM tool. Qualitative interviews were conducted by telephone or video call with a sample of stakeholders involved in adapting and implementing the tool in the local CCG area. Survey findings were summarized as frequencies and percentages. Content analysis was conducted on qualitative data using framework analysis, and data were mapped directly to the Theoretical Domains Framework (TDF).

**Results:**

Overall, 23 clinicians completed the survey, including first-contact physiotherapists (11/23, 48%), physiotherapists (7/23, 30%), specialist physiotherapists (4/23, 17%), and a general practitioner (1/23, 4%). Eight stakeholders involved in commissioning, adapting, and implementing the SDM tool were interviewed. Participants described barriers and facilitators to the adaptation, implementation, and use of the tool. Barriers included a lack of organizational culture that supported and resourced SDM, lack of clinician buy-in and awareness of the tool, challenges with accessibility and usability, and lack of adaptation for underserved communities. Facilitators included the influence of clinical leaders’ belief that SDM tools can improve patient outcomes and National Health Service resource use, clinicians’ positive experiences of using the tool, and improving awareness of the tool. Themes were mapped to 13 of the 14 TDF domains. Usability issues were described, which did not map to the TDF domains.

**Conclusions:**

This study highlights barriers and facilitators to adapting and implementing tools from one health context to another. We recommend that tools selected for adaptation should have a strong evidence base, including evidence of effectiveness and acceptability in the original context. Legal advice should be sought regarding intellectual property early in the project. Existing guidance for developing and adapting interventions should be used. Co-design methods should be applied to improve adapted tools’ accessibility and acceptability.

## Introduction

### Background

Osteoarthritis is one of the leading causes of pain and disability worldwide [1,2]. Knee osteoarthritis accounts for nearly four-fifths of the burden of osteoarthritis internationally [3]. In the United Kingdom, approximately 10% of adults have the condition [4]. Osteoarthritis is a long-term condition that does not necessarily deteriorate with time and age [5]. For most patients, pharmacological and nonpharmacological interventions are effective for managing symptoms and improving function [6]. Clinical guidelines recommend that all patients with osteoarthritis should be provided with information, education, exercise, physiotherapy, weight loss advise (as appropriate), and medication. Where these more conservative treatments are unsuccessful in alleviating symptoms, knee replacement surgery is recommended [5].

Total knee replacement (TKR) surgery can be an effective procedure for severe knee osteoarthritis [7,8]. However, it includes risks, with approximately 20% of the patients experiencing ongoing pain or dissatisfaction after knee replacement [9]. There are concerns that TKR is both over- and underused [10], evidenced by increasing provision of TKR surgery in the United Kingdom [11-14], inappropriate referrals [15], and inequity in access to surgery across different geographical regions and social groups [16,17]. The referral rates for TKR are also higher than the uptake of the most strongly recommended nonsurgical treatments (eg, weight loss and exercise) [18,19]. Both patient and clinician factors have been found to influence the over- and underuse of TKR. Patients with knee osteoarthritis have described a limited provision of information about recommended nonsurgical treatment options [20,21] and having a high expectation that they will receive surgery for their condition [22]. Clinicians report that they have inadequate educational materials on alternatives to surgery to share with patients [23] and do not have the time to provide up-to-date information on alternatives to surgery or to address patient values and preferences for treatment [19].

Shared decision-making (SDM) has been proposed in the National Health Service (NHS) Long Term Plan [24] as a way to support patients to make more informed choices about their care while simultaneously reducing inequities in access to treatment [25]. The patient is supported by the clinician to weigh up the risks and benefits of each treatment based on evidence-based information in the context of their preferences and values [26-28]. SDM is well suited to treatment decisions about knee osteoarthritis because decisions are sensitive to preference, and there are several options, each with its own risks and benefits [16]. There is evidence that SDM and the active participation of patients in treatment decision-making can improve clinical and psychosocial outcomes [29-32]. The use of decision-making tools has been found to reduce the uptake of surgery and increase the uptake of more conservative treatment options for several conditions [33-35], including knee osteoarthritis [36-38].

### SDM Tool for Knee Osteoarthritis

In 2020, the local clinical commissioning group (CCG) commissioned an SDM tool for knee osteoarthritis from another health service in the United Kingdom. Subsequently, a team of musculoskeletal commissioners, academics, and clinicians involved in treating patients with knee osteoarthritis made adaptations to the tool so that it would serve the needs of patients in the local CCG area. The tool aims to prepare patients and clinicians for SDM by supporting them to make informed choices about treatment options and improve patient-centered care planning. The tool is intended to be implemented across all points on the musculoskeletal pathway from primary to secondary care and from early- to late-stage osteoarthritis. It was anticipated that clinicians (general practitioners [GPs], physiotherapists, and surgeons) working across the pathway would use the tool with all patients, regardless of the levels of severity of knee osteoarthritis. The adapted tool was launched in the local CCG area on March 8, 2022. We evaluated the experience of the team adapting the SDM tool and the implementation potential of the tool in the local area. We took a flexible solution-focused approach to this project, shifting from a qualitative study to participatory methodology to adapt to the changing landscape of the NHS while still providing valuable findings to inform the future development of the tool.

## Methods

### Intended Use of the SDM Tool in the Local Area

The SDM tool was available in web-based and paper versions. Both versions of the tool consisted of a page of instructions for the patient describing how to use the tool, a *Helping you decide* page that gave patients suggestions about how to prepare for future appointments, a visual diagram, and written information on treatment options. It was anticipated that the web-based version of the tool would predominantly be used by clinicians and patients, with the paper version being an option to be printed out for patients who struggle with digital literacy and digital access. When the user accesses the web-based tool for the first time, they are offered a guided tour. The visual diagram consists of a table that was designed to help the patient determine their osteoarthritis stage, from early to late. The treatment options are described under the headings *Self-help*, *Non-surgical options*, *Surgery*, and *Treatments not provided by the NHS*. The position of the treatments in the table shows which treatments are appropriate for each stage of osteoarthritis. The patient or clinician can then select the treatments they are interested in and click through for further details. Details for each treatment include the following subsections: *What is it?*, *Benefits*, *Risks*, *What happens if no treatment is done?*, and *Information about support available*.

When the tool was launched, clinicians were sent guidelines on how to use the SDM tool and a copy of the paper version of the tool. The guidelines included a link to the web-based version of the SDM tool; suggestions for methods to disseminate it to patients through sharing the link in an Accurx text [39] or in appointment letters or emails; the suggestion that when prior dissemination is not possible (eg, the reason for the appointment is not known ahead of time), the SDM tool could be introduced and shown in the consultation and discussed at a follow-up appointment after the patient has had a chance to look at their options in detail; instructions on how the patient can access and use the tool; and information about how to activate and use an SDM tool EMIS template.

### Implementation Timeline for the SDM Tool

From December 2020 to March 2021, the local CCG conducted 2 one-hour and 6 three-hour SDM training sessions, at the end of which a questionnaire was sent to all attendees asking whether they were willing to pilot the SDM tool. Attendees included GPs, physiotherapists, orthopedic surgeons, and occupational therapists.

The SDM tool was planned to be launched *officially* in September 2021. However, this was delayed until March 2022 owing to the negotiation of formal collaboration agreements among the stakeholder organizations.

In January 2022, the branding of the adapted tool was agreed, and collaboration agreements were finalized, allowing it to be officially launched in March 2022.

### Design

In describing our approach, we have adhered to the COREQ (Consolidated Criteria for Reporting Qualitative Research) guidelines on the reporting of qualitative research [40]. Originally, we had intended to interview up to 20 patients, 20 clinicians, and 5 commissioners about their experience of developing (commissioners) or using (patients and clinicians) the tool. The interviews with the commissioners were planned as patient and public involvement (PPI), whereas the interviews with the patients and clinicians were planned as qualitative research. Because of staff pressures imposed by the COVID-19 pandemic, the recruitment of clinicians and patients was substantially hampered. After discussion with key stakeholders and the study sponsor (University of Bristol), the study design was amended to become a partnership approach using mixed methods [41]. This included (1) a web-based survey to elicit feedback on the SDM tool from clinicians with free-text and multiple-choice responses and (2) a further 3 interviews for the PPI component to explore the views and experiences of the team involved in developing and implementing the tool with the aim of understanding its acceptability and views on its implementation potential. The University of Bristol PPI team provided guidance that anonymized quotes could be used in the dissemination of the work, where consent had been obtained from the people involved in the survey and interviews.

### Ethics Approval

Ethics approval was granted for the original research design by the West of Scotland REC 5 research ethics committee on the 22nd of December 22, 2021 (21/WS/0163). However, ethical approval was not sought for the final research design which used a partnership approach, as it was considered to be PPI.

### Methodological Approach

The methodological orientation used for the interviews was a deductive approach to content analysis [42]. The theoretical underpinning for this qualitative study was the Theoretical Domains Framework (TDF) [43]. The TDF provides a theoretical basis for implementation studies and supports the identification and interpretation of barriers and facilitators to the implementation of interventions in practice [43]. The TDF was developed by a team of behavioral scientists and implementation researchers through a consensus process [44], with 12 domains incorporating 128 theoretical constructs from 33 theories that were judged to be most relevant to implementation questions. After a validation exercise, a 14-domain version of the TDF was developed (version 2.0), which covered 84 theoretical constructs. Version 2.0 has been used in this study [45]. The 14 domains cover individual motivation and capability, as well as factors in the physical and social environment [43].

### Participants

Clinicians were invited to complete the survey (Multimedia Appendix 1) through snowballing information from musculoskeletal clinical team leaders across primary and secondary care services within the CCG, as well as our research partners in the local NHS Trust and health care providers. Participants were selected for one-on-one interviews if they had been involved in commissioning the SDM tool or adapting the tool for the local area. A purposive sample was sought for the interviews in terms of the role in commissioning the tool for musculoskeletal conditions, adapting the tool, type of clinician, and role in implementation.

### Procedure

The survey was developed in the Jisc online survey system [46]. ST, AJM, and NEW were involved in the development of the survey. The survey was deliberately kept very simple because the work was conducted during the COVID-19 pandemic when restrictions were in place. Because of the simplicity of the tool and the necessity for us to be responsive within the short time frame, no formal validation was conducted. The results were stored on secure University of Bristol servers. A link was circulated via email to potential participants and was available from May 17, 2022, to June 30, 2022. The participants were informed that the purpose of the survey was to evaluate the implementation of the SDM tool. They were informed that providing a response amounted to consent to participate in the study and were asked whether they agreed to the use of anonymized responses in subsequent publications.

Potential participants for the semistructured interviews were approached directly by email and offered a telephone or video call at a time that suited them. At the beginning of each interview, participants were given the opportunity to ask questions and asked for their consent to the interview being audio recorded and to their anonymized quotes being published. Two topic guides were developed, one for commissioners involved in adapting and implementing the tool (Multimedia Appendix 2) and another for those who were using it in their clinical practice (Multimedia Appendix 3). The topic guides were developed by the research team and collaborators and were based on elements from the TDF to include factors that might influence the use and implementation of the SDM tool [39]. The interviews were conducted by 1 researcher (ST) and transcribed verbatim. Field notes were taken during and after the interviews.

### Analysis

For the survey findings, categorical variables were summarized as frequencies and percentages. Interviews were audio recorded with participants’ consent, transcribed, anonymized, and checked for accuracy before being uploaded to NVivo qualitative data management software (release 1.6.2; Lumivero) [47]. Data collection and analysis were iterative and immediate to allow emerging issues to be explored in subsequent interviews. Interviewing continued until we had developed a sufficient level of information power [48], which enabled us to address the aims of the study in accordance with its narrow focus on barriers and facilitators to implementation and the inclusion of a highly specific group of participants.

We followed the guidelines provided in the study by Aitken et al [43] to conduct deductive analysis of qualitative data using the TDF. For both the free-text responses from the survey and the qualitative interviews, content analysis was conducted using the TDF to generate a coding framework [49,50], with quotes being mapped directly to the 14 domains and subdomains outlined in the TDF (version 2.0) [45]. Many of the same themes were coded to multiple domains of the TDF. Therefore, to avoid repetition and to improve the interpretation of our results, we present details of the themes and indicate which domains of the TDF the themes were coded to in the text and in Multimedia Appendix 4. We have also included illustrative quotes for the themes in Multimedia Appendix 4.

Transcripts were read by 1 researcher (ST), who considered the responses in relation to the TDF domains. ST subsequently coded the participant responses to ≥1 of the 14 theoretical domains of the TDF, assigning them to constructs within the domains [43]. A sample of the interview transcripts were read independently by AM and discussed at research team meetings with ST and NW to ensure that the data were coded into the appropriate domains of the TDF and that any subjectivity was reduced. An inductive approach was also used to code responses that did not fit within the TDF domains to ensure that important issues were not overlooked or excluded [51]. Participants were provided with a summary of the findings; none of them responded with feedback.

### Research Team and Reflexivity

#### Personal Characteristics

ST is a mixed methods postdoctoral researcher with a background in psychology, whose research focuses on the development and evaluation of health interventions and on how to make access to these interventions equitable. ST believes that digital tools can be beneficial to different patient groups but are often developed and disseminated in a way that increases inequalities owing to issues with accessibility and usability. ST’s qualifications include PhD, MSc, and BSc degrees. AJM, who conceptualized the study, is a senior lecturer in musculoskeletal health services research and a qualitative methodologist specializing in research on improving the management of osteoarthritis and orthopedic outcomes. NW is an academic physiotherapist and professor of knowledge mobilization with expertise in musculoskeletal research.

#### Relationship With Participants

ST has attended meetings with several of the interview participants but did not have a working relationship with them. The interview and survey participants knew that the study was evaluating the implementation and use of the SDM tool.

## Results

### Overview

The qualitative data were grouped according to themes, and the TDF domains are highlighted in Multimedia Appendix 4 and throughout the text. The themes were related to barriers and facilitators to the development of the SDM tool, use of the tool for clinicians and patients, and implementation of the tool. The interview durations ranged from 15 to 61 minutes, with a mean of 37 minutes.

### Sample

#### Survey

Overall, 23 clinicians responded to the survey: first-contact physiotherapists (11/23, 48%), physiotherapists (7/23, 30%), specialist physiotherapists (4/23, 17%), and a GP (1/23, 4%). The majority have been in their role for 1 to 2 years (11/23, 48%; Table 1). Of the 23 clinicians, 5 (22%) did not consent to having anonymized quotes published.

**Table 1:** Survey sample (N=23).

		Values, n (%^a^)
**Professional role**		
	First-contact physiotherapist	11 (48)
	Physiotherapist	7 (30)
	Specialist physiotherapist (extended scope, orthopedic, and advanced physiotherapy practitioner)	4 (17)
	GP^b^	1 (4)
**Time in role (years)**		
	<1	3 (13)
	1-2	11 (48)
	3-5	1 (4)
	6-10	3 (13)
	11-15	0 (0)
	>15	5 (22)
**Role in delivering the SDM^c^ tool**		
	Involved in managing or overseeing the SDM tool	0 (0)
	Involved in delivering the SDM tool	21 (91)
	Other (asked to try the SDM tool)	2 (9)

^a^May not add up to 100% owing to rounding.

^b^GP: general practitioner.

^c^SDM: shared decision-making.

#### Interviews

Overall, 8 health care professionals were interviewed, all of whom consented to having anonymized quotes published. Participants were selected on the basis of being involved in commissioning or delivering care for musculoskeletal conditions and in the development or dissemination of the SDM tool. No potential participant declined to be interviewed. Of the 8 participants, 3 (38%) described a role exclusively in the development of the tool (interviewees A, B, and C): interviewee A was involved in improving the look and layout of the paper version of the tool, interviewee B was involved in clinical content development, and interviewee C had been involved in delivering SDM training and advising on the accessibility of the tool. Interviewee D was not involved in the development or implementation of the tool but described how their role as a director meant that they were involved in creating an environment that would support the development of SDM tools. Interviewee E spoke about their role as a program manager and the ways in which they supported the development and implementation of the SDM tool, including applying to become an SDM accelerator site, commissioning the SDM tool, applying for funding to support the project, arranging contracts, liaising with the digital development provider, and bringing together clinical and academic collaborators to develop the tool. Of the 8 participants, 3 (38%) were involved in the development of the tool, led on dissemination in their service, and were using the tool in their practice (interviewees F, G, and H). Of these 3 participants, 2 (67%) were physiotherapists (interviewees F and G), and 1 (33%) was an orthopedic surgeon (interviewee H).

### Quantitative Survey Findings

Table 2 provides a summary followed by a description of the quantitative survey findings.

**Table 2:** Summary of quantitative survey findings.

			Values, n (%^a^)
**How did you come to know about the SDM^b^ tool? (N=23)**			
	Managers or clinical leads	5 (22)	
	Training session or team meeting	7 (30)	
	Employer	6 (26)	
	Colleagues (emails and meetings)	4 (17)	
	“When this survey was sent to me”	1 (4)	
**What is your role in relation to the tool? (N=23)**			
	I am involved in managing or overseeing the [SDM tool]	0 (0)	
	I am involved in delivering the [SDM tool]	21 (91)	
	Other (asked to try the tool)	2 (9)	
**Have you ever used the tool in your practice? (N=23)**			
	Yes	17 (74)	
**How many times have you used or recommended it to your patients? (n=17)**			
	1	0 (0)	
	2-5	11 (65)	
	6-10	4 (24)	
	11-15	1 (6)	
	16-20	0 (0)	
	>20	1 (6)	
**How do you access it? (eg, using a link sent to you/on [local referral system]; n=17)**			
	Most had accessed the tool via a web link	13 (76)	
	Bookmarked the website address	1 (6)	
	Googling the name of the tool and clicking on the first link	1 (6)	
	“Via the website”	1 (6)	
	“The internet”	1 (6)	
**Could you tell us how you have used it in your practice? (eg, sending it to patients to read in their own time/showing them the tool during the consultation; n=17)**			
	Discussed the tool during a consultation only	5 (29)	
	Discussed the tool during a consultation...and signposted patient to the tool using a link	5 (29)	
	Discussed the tool during a consultation...and printed out paper version	1 (6)	
	Discussed the tool during a consultation...and showed them information about the tool on a poster in their clinical space	1 (6)	
	Sent link to the patient without discussing it in the consultation	4 (24)	
	Sent link to the patient without discussing it in the consultation...but then discussed the tool in a follow-up consultation	1 (6)	
**Do you plan to use or recommend the tool to your patients in the future? (N=23)**			
	Yes	19 (83)	
**Have you had any challenges when accessing or using the [** **SDM** **tool]? (n=22)**			
	Yes	15 (68)	
	No, happy with the existing tool	2 (9)	
	Had not used the tool enough to comment	5 (23)	

^a^May not add up to 100% owing to rounding.

^b^SDM: shared decision-making.

#### Hearing About the SDM Tool

The clinicians had heard about the SDM tool from their managers (5/23, 22%), employers (6/23, 26%), colleagues (4/23, 17%), and at training sessions or team meetings (7/23, 30%). Of the 23 clinicians, 1 (4%) reported hearing about the tool only when the survey was sent to them.

#### Clinicians’ Engagement With the SDM Tool

Most of the respondents (21/23, 91%) were involved in the delivery of the SDM tool, and 2 (9%) of the 23 respondents reported that they were asked to try the tool. Nearly three-quarters (17/23, 74%) of the clinicians had used the SDM tool, the majority of whom (11/17, 65%) had used it 2 to 5 times. Most of the respondents (13/17, 76%) had accessed the tool via a web link. Others had bookmarked the website address (1/17, 6%), googled the name of the tool and clicked on the first link (1/17, 6%), accessed it “via the website” (1/17, 6%), or found it on “the internet” (1/17, 6%).

#### How Clinicians Used the SDM Tool

Most of the clinicians (12/17, 71%) who used the SDM tool described showing the tool to, or discussing it with, the patient during a consultation. Some of these clinicians then signposted the patient to the tool using a link (5/12, 42%), printing out the paper version (1/12, 8%), or showing them information about the tool on a poster in their clinical space (1/12, 8%). Of the 17 clinicians, 5 (29%) described sending the link to the patient without discussing it in the diagnostic consultation. However, of these 5 clinicians, 1 (20%) said that they would then discuss the tool in a follow-up consultation.

#### Future Use of the Tool and Improvements

Of the 23 clinicians, 19 (83%) reported that they planned to recommend the SDM tool to their patients in the future. Of these 19 clinicians, 9 (47%) recommended improvements, and 5 (26%) said that they had not used it enough to be able to recommend changes.

The clinicians were asked whether they had any issues accessing or using the SDM tool. Of the 22 clinicians who responded to this question, 15 (68%) provided details of the issues they had experienced (detailed in the *Qualitative Findings From the Survey and Interviews* subsection). Other clinicians reported no issues and felt happy with the current version of the tool (2/22, 9%) or had not used the tool enough to comment (5/22, 23%).

### Qualitative Findings From the Survey and Interviews

#### Overview

There were 8 overarching themes that described the barriers and facilitators to the adaptation of the SDM tool, implementation of the tool, use of the tool by clinicians, and use of the tool by patients. These themes mapped to 13 of the 14 TDF domains, with no quotes mapping to the *behavioral regulation* domain (Multimedia Appendix 4). The most salient domains were the *environmental context and resources* and *social or professional role and identity* domains. Participants also described usability issues with the SDM tool and improvements that could be made, which did not map to any of the TDF domains.

#### Barriers to the Development and Adaptation of the SDM Tool

##### Conflicting Priorities and Perspectives of the Developers of the Original Tool and the Team Adapting the Tool for the Local Area

There were some disagreements resulting in late negotiations between the needs of the team that developed the original tool and the team that adapted it for the local CCG area (*environmental context and resources*; *memory, attention, and decision processes;* *intentions*; and *goals* domains). This slowed the development of the tool and limited the adaptations that could be made. Those who adapted the tool had felt that the original tool needed to be updated to include the most up-to-date best evidence (*knowledge* domain). They also spoke about how adaptations were limited because the innovator wanted to retain elements of the original tool, had changed their mind about how much the tool could be altered, and had strong views on which digital development company should host the tool.

The program manager (interviewee E) described how formulating agreements about the intellectual property (IP) of the SDM tool slowed down the release of the adapted tool in the local area (*environmental context and resources* domain). They spoke about having a lack of knowledge about IP arrangements (*knowledge* domain), and consequently, they started the project with the belief that the different collaborating organizations could work together without using lawyers to draw up contracts to resolve IP issues (*optimism* domain).

##### Perceived Pressure to Complete and Release the SDM Tool Owing to Time and Budget Restrictions in the CCG

Some of the interview participants questioned whether time and financial pressures in the CCG resulted in the tool being released prematurely, which affected the quality of the SDM tool (*environmental context and resources*; *goals*; and *memory, attention, and decision processes* domains). Some of the participants suggested that it might have been better to create a new tool rather than adapt an SDM tool from another area. At the time this was not presented as an option because the money had already been spent on procuring the tool.

##### Conflicting Time Demands and Clinical Perspectives of the Collaborators in the CCG

Most of those involved in the adaptation of the SDM tool for the local area spoke about how they had to work on the tool in their spare time and were not provided with protected time to work on the project (*environmental context and resources* domain). They also described the challenges of combining their different clinical perspectives when adapting the tool (*social or professional role and identity* domain).

##### Being Limited by How Many Technical and Visual Adaptations Could Be Made to the Original Tool

There were frustrations about being *tethered* to the design of the original tool and about the technical limitations with regard to the adaptations that could be made to the tool by the digital developers, which the team felt prevented them from developing something that could potentially work better for the local area (*environmental context and resources* domain).

#### Barriers to Implementation

##### SDM Not Being a Defined and Resourced Priority for the NHS

The interview participants felt that SDM was not defined as an NHS priority, resulting in a lack of resources being allocated to support it (*environmental context and resources* and *goals* domains). Several commissioners spoke about the need for the NHS to invest resources to maintain and update the SDM tool (*environmental context and resources* domain). There were also concerns about what would happen to the SDM tool when the musculoskeletal project manager left their post, and the CCGs were abolished and replaced with integrated care boards.

##### Clinical Leaders Did Not Have Buy-In

Some participants in leadership positions were happy to support the dissemination of the SDM tool (*social or professional role and identity* domain), although others did not feel that they could support the final iteration of the tool because they were concerned about its quality (*beliefs about consequences* domain). This concern stemmed from challenges experienced during the adaptation process where issues with version control meant that the tool had been launched without incorporating all agreed changes. Another participant involved in the adaptation of the tool reported being unconvinced about the tool’s potential to help patients (interviewee A, *optimism* domain).

#### Barriers to the Use of the Tool for Clinicians

##### Limited Awareness of the Tool Among Clinicians

In the interviews and survey, participants indicated that other clinicians were not widely aware of the SDM tool and its purpose, inhibiting its uptake (*knowledge* domain). They believed that this was due to a lack of a more considered dissemination strategy during the launch of the tool coupled with ongoing technical issues, which limited the spread of the tool (*environmental context and resources* and *knowledge* domains).

##### Challenges With the Web Address and Finding the Web-Based Tool by Web Search

Interview and survey participants described issues with finding the local CCG version of the SDM tool through web-based searches and through the CCG’s website (*environmental context and resources* and *knowledge* domains). This was caused by issues with the website address and an absence of search optimization.

##### Usability Issues With the SDM Tool (Not Mapped to the TDF Domains)

Some of the survey participants (2/23, 9%) felt that navigating to or around the web-based version was challenging. Others (5/23, 22%) felt that the paper version was too large to use, which presented a barrier to printing out paper copies in the clinic.

##### Perception That the SDM Tool Was Not as User-Friendly as Other SDM Tools

Participants in the interviews and survey felt that other nationally available SDM tools were easier and simpler to use and planned to use them rather than the newly adapted SDM tool. These tools could also be printed out easily because they were only a few pages long (*intentions* and *beliefs about consequences* domains).

##### Limited Time to Discuss the Tool in Consultations

Several of the clinicians (6/23, 26%) felt that there was not enough time to use the tool in a patient consultation (*environmental context and resources* domain). Whereas some of the clinicians showed the tool to patients or mentioned it in the consultation (12/17, 71%), others sent a link to the SDM tool after the session (5/17, 29%). A survey participant felt that sending the SDM tool to the patient after the appointment could potentially result in the need for additional appointments to discuss the treatment options described (*environmental context and resources* domain).

##### Clinicians Having to Remember Multiple Tools

Some of the interview participants described how adding to a plethora of tools that clinicians and patients have to manage and remember could potentially limit the uptake of the SDM tool (*memory, attention, and decision processes* domain).

##### General Negative Perceptions of SDM Tools

Interviewees D and H described their belief that orthopedic surgeons had generally negative views of SDM tools because they believed that the NHS was using them to save money and resources by reducing the number of surgeries (*social or professional role and identity* and *beliefs about consequences* domains).

#### Health Care Professionals’ Beliefs About Potential Barriers to Patients Using the SDM Tool

##### Accessibility Issues

Several of the interview participants were concerned that patients may have issues accessing and using the SDM tool if their first language was not English, they lacked digital skills or digital access, or they had low health literacy (*environmental context and resources* domain). Consequently, some of the participants did not plan on implementing the SDM tool in their practice (*goals* domain). A clinician also described how they believed that some cultural groups expect a more didactic type of medicine, which contrasts with the SDM approach (*social influences* domain).

##### Need for Mobile Optimization

Several of the interview participants felt that the lack of a mobile app version of the tool would be a potential barrier that patients might experience (*environmental context and resources* domain). However, a physiotherapist felt that the SDM tool did not need to be optimized for mobile phone use because older adults were more likely to engage with digital support on iPads or laptop computers (interviewee F; *beliefs about capabilities* domain).

#### Facilitators to the Adaptation of the SDM Tool

##### Skills of Collaborators

Some of the interview participants felt that the collaboration between academic researchers and clinicians from across the care pathway (including GPs, physiotherapists, and surgeons) in the adaptation process had led to improvements to the original tool, particularly in terms of ensuring that its content was evidence based (*social or professional role and identity* and *skills* domains).

##### Group Identity

A commissioner suggested that it was important that those who had contributed to the development of the original and adapted versions of the tool felt that they are part of a team, that this would ensure that the adaptation of the tool could continue (*social or professional role and identity* domain), and that it could continue to be disseminated across different regions in England.

##### Accessing Funds to Adapt the SDM Tool and Make it More Accessible

The program manager described how they had applied for funding to produce videos to embed in the tool and translate the tool into different languages to improve accessibility (*environmental context and resources* domain). They also described the important role that NHS funds played in paying a local university for a researcher’s time on the project to ensure that the content was evidence based.

#### Facilitators to Implementation

##### Organizational Culture Supports SDM

A commissioner described how they were using their leadership role to reinforce the message that SDM is useful and felt that this would support SDM tools “to grow” (interviewee D; *environmental context and resources* and *social or professional role and identity* domains).

##### Access to Resources to Support SDM in the Local Area That Supported the Commissioning of the Tool

Interview participants spoke about how NHS initiatives, training, and grants had supported SDM in the local area as well as the commissioning and adaptation of the SDM tool (*environmental context and resources* and *skills* domains).

##### The Importance of Demonstrating Evidence of Improved Patient Outcomes and Resource Use in the Local Area

Some of the interview participants felt that the tool would be maintained if there was evidence that it was acceptable to users and could be shown to improve patient outcomes and resource use in the NHS (*environmental context and resources* and *beliefs about consequences* domains). They felt that this would support appeals for other clinicians to use the tool, attract more resources to develop the tool and SDM more generally, and encourage collaborators to continue to develop the tool (*reinforcement* and *beliefs about consequences* domains).

##### Clinicians and People in Leadership Positions Believe That SDM Tools Can Improve Patient Outcomes and Reduce Resource Use

Several of the interview participants felt that the adapted SDM tool had the potential to improve outcomes for patients and reduce the pressure on the health service (*optimism* domain). Participants spoke about the potential of the adapted SDM tool to develop patients’ self-care skills, provide evidence-based information and treatment options to support SDM, and help patients to make informed decisions about their care (*skills*, *knowledge*, and *beliefs about consequences* domains). They felt that it was valuable having a resource that would support patients to remember what was discussed in the consultation (*memory, attention, and decision processes* domain) and their subsequent management decisions (*beliefs about consequences* domain).

##### People in Leadership Positions Were Motivated to Increase Awareness of and Engagement With the SDM Tool

Some of the interview participants spoke about the efforts they had made to increase the uptake of the adapted SDM tool, such as spreading the word about the tool to other clinicians (*knowledge* and *social or professional role and identity* domains).

##### Suggested Improvements to Where in the Care Pathway the SDM Tool Is Delivered

Clinicians responding to the survey were asked what improvements could be made to the adapted SDM tool, and some of them (5/23, 22%) thought that the SDM tool would be most useful if it was sent to patients before the diagnostic consultation and promoted earlier in the primary care pathway (*knowledge* and *goal priority* domains).

#### Facilitators to the Use of the SDM Tool by Clinicians and Patients

##### Clinicians Had Positive Perspectives and Experiences of Using the SDM Tool

When asked which aspects of the SDM tool they found most useful, some of the survey participants (9/17, 53%) described how it was helpful to have a (visual) timeline, explaining the stages of disease and appropriate recommended management options for each stage (*knowledge* domain). Others (8/17, 47%) said they found it useful to have all of the management and treatment options summarized concisely in one location. Some (2/17, 12%) felt that this supported SDM and helped to guide patients in their decisions about what treatments might be best for them (*goals* domain). Others (2/17, 12%) also found the specific information on risks and benefits useful (*beliefs about consequences* domain).

##### Making Improvements to the Content and Usability of the SDM Tool (Not Mapped to the TDF Domains)

Survey participants were asked for suggestions for how to improve the SDM tool. They felt that usability could be improved through simplification and instructions for how to use it, as well as by making the information more concise. Of the 23 participants, 5 (22%) felt that it would be useful to have a paper version available that they could print out on 1 or 2 pages. A commissioner felt that patients needed clearer messaging about what the tool was and how they could use it.

##### Using Existing Health Care Communication Systems (eg, Accurx Templates, NHS Referral Website, and Letters to Patients) to Support the Visibility and Use of the SDM Tool

Interview participants felt that existing NHS dissemination routes needed to be used to increase awareness of the SDM tool among clinicians (*environmental context and resources* domain), for example, embedding links in Accurx templates, having the tool visible on the local CCG referral system, and sending a link to the web-based tool through the GP practice SMS text message systems to the appropriate patients.

##### Active Approaches to Increasing Awareness of the SDM Tool Among Patients

Interview and survey participants described different approaches to using the SDM tool with their patients. They either used it actively during the consultation or by sending a link to the patient before or after the consultation (*knowledge* and *social or professional role and identity* domains). A commissioner felt that the SDM tool would be most useful if used in conversation with clinicians rather than handed to the patient to work through on their own (*environmental context and resources* domain).

## Discussion

### Principal Findings

This study aimed to explore the experience of a team adapting an SDM tool for knee osteoarthritis from one health service to another and its implementation potential in the local CCG area. Study participants described barriers and facilitators to both the adaptation of the SDM tool and its implementation and use by clinicians and patients. Facilitators included features of the tool that described treatments appropriate to the patient’s stage of disease, proactive use of the tool, sending it to patients before consultations, using existing NHS dissemination routes to increase awareness of the tool among clinicians, and increasing awareness through clinical leaders and champions. Participants also suggested improvements to the tool’s design and dissemination strategies that could increase its implementation potential and uptake in the future. These themes were mapped to 13 of the 14 TDF domains, with the most salient domains being *environmental context and resources* and *social or professional role and identity*. Participants also described usability issues with the SDM tool and improvements that could be made, which did not map to the TDF domains.

### Comparison With Prior Work

Our findings concur with those of Légaré et al [52], whose systematic review of barriers and facilitators to SDM showed that SDM in clinical practice was facilitated by the motivation of clinicians and their perception that SDM would improve patient outcomes and health care processes. The systematic review also reported that across organizational and cultural contexts, the most highly cited barrier was time restraints, followed by a perceived lack of applicability of SDM owing to patient characteristics or clinical situation [52]. In our study, participants often referenced time restraints in their practice. Some of the clinicians interviewed also felt that patients’ engagement with the SDM tool would be affected by particular characteristics, for example, those who were not fluent in the English language or who had lower digital and health literacy levels. The authors of the systematic review cited concerns that these perceptions about which patients would respond positively to SDM and associated tools would result in clinicians only using the tool with selected patients whom they felt might benefit, which would create inequalities in access to SDM and SDM tools [52].

Several suggestions have been made in the literature about how to overcome the challenges of implementing SDM and SDM tools in the NHS [26]. These include making patient decision support available before referral [26], adapting tools rather than reinventing them [26], making tools accessible to both patients and clinicians [53], ensuring that they fit into clinical workflows [53], and having buy-in from the health organization [54]. They suggest that tools that provide short summaries for use in clinical encounters alongside longer sources of information for patients to read in their own time may be more readily adopted by clinicians [26,54-57]. These mirrored suggestions from our participants about how to improve the implementation, use, and sustainability of the SDM tool in the long term.

### Strengths and Limitations

We took a flexible solution-focused approach to this project, responding to challenges we faced in recruiting participants and a rapidly changing landscape in the NHS by shifting the methods from traditional qualitative research to a participatory approach. We were unsuccessful in recruiting clinicians or patients for interviews as originally planned when advertising the study through clinical networks and musculoskeletal clinical groups. This is unfortunate because it limits the breadth of perspectives from different stakeholders. When we investigated possible reasons for the poor response, clinical leads reported that clinicians were under severe time pressures after the COVID-19 pandemic and were unable to spare time to be interviewed or to recruit patients for the study. After discussion with the study sponsor, the study design was amended to include interviews with commissioners and team members involved in the adaptation and implementation of the tool and a short web-based survey that could be completed quickly by clinicians. We consulted with the project sponsors and the PPI team at the University of Bristol to consider ethical issues around the publication of findings from this partnership approach [58] and sought approval to publish anonymized quotes, which allowed us to provide rich descriptive data to illustrate our findings.

The survey was disseminated widely through the CCG area by snowballing information from musculoskeletal clinical team leaders across primary and secondary care services within the CCG, as well as our research partners in the local NHS Trust and health care providers. Of the 23 survey respondents, 22 (97%) were physiotherapists who had engaged and had had positive experiences with the tool. We may therefore have missed capturing the views of those from different clinical backgrounds and those who were less engaged with the tool. We only received surveys that had been completed; therefore, we do not have additional information about those who may have declined to complete it. The low response rate is also likely due to time pressures after the COVID-19 pandemic. We recognize that the timing of implementation was not ideal, given the huge pressures on the health care system at the time.

### Implications for Future Research, Policy, and Clinical Practice

This study has implications for future commissioning and adaptation of tools for different regions of the NHS. There were elements of good practice highlighted in this study. The team in the local CCG that commissioned the SDM tool followed the recommendation to adapt an existing tool rather than using resources to develop a new one [26]. The CCG team involved a multidisciplinary team to adapt the tool to ensure that the information was evidence based and would support clinicians and patients to make decisions across the care pathway for knee osteoarthritis [59]. The CCG team also sought to evaluate adaptations made to the tool in this project [59]. However, there were limited resources available to the team that adapted the tool, as well as a lack of specialist intervention development expertise. We would make the following recommendations for teams involved in commissioning and adapting tools from one context to another (many of these are also highlighted in the ADAPT guidance on how to adapt interventions to new contexts [59]):

- When selecting a tool to be adapted for a new context: ensure that the tool selected for adaptation has a strong evidence base and good evidence of effectiveness and acceptability to the target users (clinicians and patients) in the original context [59].
- When commissioning new tools for adaptation: seek out legal advice at the beginning of the project and draw up agreements among collaborators to ensure that all parties are clear on expectations, IP, and any limitations to the changes that can be made.
- When adapting the tool: ensure that there is expertise represented in adapting and developing behavior change and SDM tools. The experts should have knowledge and experience of using appropriate methodologies to ensure that the tool will be acceptable and effective in the new context. This includes drawing on Medical Research Council guidance for the development and evaluation of complex interventions [60] as well as the ADAPT guidelines [59]. Normalization process theory can be applied to ensure that interventions can be normalized into routine clinical practice, thus increasing implementation potential and acceptability to clinicians [61]. The accessibility and acceptability of the tool can be improved using co-design methods. An example would be the person-based approach, which considers and addresses the needs of users and potential barriers to engagement with the tool [62].

Researchers may also consider the use of other tools, such as the theoretical framework of acceptability, to support the assessment of the acceptability of health interventions earlier on in the co-design process [63].

### Conclusions

This study highlights the barriers and facilitators to adaptation of an SDM tool from one health context to another and its implementation. We recommend that teams involved in commissioning and adapting tools from one context to another should ensure that the tool selected for adaptation has a strong evidence base as well as evidence of effectiveness and acceptability to the target users in the original context. Teams commissioning the tool should seek out legal advice at the beginning of the project and draw up agreements among collaborators to ensure that all parties are clear on expectations and aware of any limitations to changes that can be made. Existing guidance for the development and adaptation of interventions should also be used early on in the process (eg, Medical Research Council and ADAPT guidelines as well as normalization process theory). The accessibility and acceptability of the tool can be improved using co-design methods.

## Appendix

Multimedia Appendix 1 Survey for clinicians who used the shared decision-making tool.

Multimedia Appendix 2 Interview topic guide for commissioners involved in adapting and implementing the tool.

Multimedia Appendix 3 Topic guide for health care professionals who used the tool.

Multimedia Appendix 4 Summary of themes with mapping to the Theoretical Domain Framework (version 2.0).

## Abbreviations

CCG: clinical commissioning group
COREQ: Consolidated Criteria for Reporting Qualitative Research
GP: general practitioner
IP: intellectual property
NHS: National Health Service
PPI: patient and public involvement
SDM: shared decision-making
TDF: Theoretical Domains Framework
TKR: total knee replacement

## Data Availability

The qualitative data sets generated and analyzed during this study are not publicly available owing to intellectual property arrangements and the sensitive nature of the interviews.
