# Supplementary material for: Adaptation and Implementation of a Shared Decision-Making Tool From One Health Context to Another: Partnership Approach Using Mixed Methods
Source: J Med Internet Res. 2023 Jul 5;25:e42551. doi: 10.2196/42551 (PMC10357316; doi:10.2196/42551)
Supplement: Multimedia Appendix 1 [file jmir_v25i1e42551_app1.pdf]

In this brief survey we will be asking for your views on the Shared Decision-Making (SDM) tool. Completing the survey should take no more than 10 minutes of your time.

The findings from the survey will be used to help researchers at the University of Bristol and partners in the [CCG] to evaluate the implementation of the tool. Please be as honest is possible. We would also like to hear from people who have not used the tool, to understand what barriers to use might be.

Many thanks in advance for your time.

If you have any questions, please contact Dr Sophie Turnbull: sophie.turnbull@bristol.ac.uk.

1. What is your role in relation to the SDM tool (tick any that apply)
    - I am involved in managing or overseeing the tool
    - I am involved in delivering the tool
    - Other (please describe)
        i. Free text box
2. How would you describe your professional job category?
        i. Free text box
3. How long have you worked in your role? (multiple choice)
    - Less than one year
    - 1-2 years
    - 3-5 years
    - 6-10 years
    - 11-15 years
    - More than 15 years
4. Would you be happy for us to publish anonymous quotes you have provided in this survey in publications and at conferences?
        i. Yes/No
5. If you would like to be provided with a summary of our findings please provide your email address below.
        i. Email address
6. How did you come to know about the tool?
        i. Free text box
7. Have you ever used it in your practice?
        i. Yes/No
    - (For those that respond No) If you haven't used it, could you tell us why?
        i. Free text box (Then jump to question 13)

For those that respond yes to 7:

8. Could you tell us how you have used it in your practice? (e.g. do you send to patients to read in their own time, or show them the tool during the consultation)
        i. Free text box
9. How do you access it? (e.g. using the link sent to you/ on Remedy)

    i. Free text box

10. How many times have you used it? (Provide a range)
    - Once
    - 2-5 times
    - 6-10 times
    - 11-15 times
    - 16-20 times
    - More than 20 times
11. What part of the tool did you find most useful?
    i. Free text box
12. How did you find recording the outcomes and use of the tool on EMIS?
    i. Free text
13. Do you plan to use or recommend the tool to your patients in the future?
    i. Yes/No
    - (For those that respond No) If you do not plan to use it in the future could you explain why?
        i. Free text box
14. Have you had any challenges when accessing or using the tool?
    i. Free text
15. Do you think anything about the tool can be improved?
    i. Free text
16. If you were only going to focus on one thing to change which would you prioritise?
    i. Free text
17. Any other comments
    i. Free text

Thank you for your time taken answering this survey. If you have any questions please contact Dr Sophie Turnbull: sophie.turnbull@bristol.ac.uk.
