# Supplementary material for: Adaptation and Implementation of a Shared Decision-Making Tool From One Health Context to Another: Partnership Approach Using Mixed Methods
Source: J Med Internet Res. 2023 Jul 5;25:e42551. doi: 10.2196/42551 (PMC10357316; doi:10.2196/42551)
Supplement: Multimedia Appendix 2 [file jmir_v25i1e42551_app2.pdf]

Introduction

- Thanks, introduce self and re-state the purpose of the interview: To explore views of the Shared decision-making (SDM) tool for knee osteoarthritis (OA).
- Discussion how the interview will be recorded, issues of confidentiality, anonymisation.
- Check they are happy for the quotes to be published.
- Remind them that it helps us if they are honest with their feedback, even if it is negative and we will not get offended. This all helps us to improve the SDM tool for the future.

Background

- What has been your experience of commissioning for Musculo-skeletal (MSK) conditions?
    - Prompt: How long have you been focussing on MSK commissioning??
- Can you tell me a little bit about shared decision making in musculoskeletal health and what sort of role or interest you've had in helping to develop it?

Avon Knee Chart

- How did you first come to hear about the SDM tool?
- Have you been involved at all in reviewing the SDM tool or developing it?
- What sort of role have you played in its development and implementation so far?
- What have been the challenges you've faced? (have these been overcome and if so, how?)
- How sustainable do you think the SDM tool is?
- Do you think the SDM tool might have any influence on value-based healthcare? (what sort of influence?)
- Do you think there's anything about the SDM tool itself or the local systems that can be improved to support its sustainability?

Conclusions

- Is there anything else you would like to talk to me about that you feel might be important that I've missed?

Thank you and check ok to finish.
