# Supplementary material for: Adaptation and Implementation of a Shared Decision-Making Tool From One Health Context to Another: Partnership Approach Using Mixed Methods
Source: J Med Internet Res. 2023 Jul 5;25:e42551. doi: 10.2196/42551 (PMC10357316; doi:10.2196/42551)
Supplement: Multimedia Appendix 3 [file jmir_v25i1e42551_app3.pdf]

Introduction

- Thanks, introduce self and re-state the purpose of the interview: To explore views of the shared decision-making (SDM) tool for knee osteoarthritis (OA).
- Discussion how the interview will be recorded, issues of confidentiality, anonymisation.
- Check they are happy for the quotes to be published.
- Remind them that it helps us if they are honest with their feedback, even if it is negative and we will not get offended. This all helps us to improve the SDM tool for the future.

Background

Could you start by telling me a bit about your experience working with patients with osteoarthritis.

- What does SDM mean to you in your practice?
- What are your views on SDM around treatment for OA (Prompt – Is this something you're comfortable with or find difficult at times?
- What are the sorts of difficulties/barriers you might face with SDM for OA?

SDM tool for Knee OA

- How did you first come to hear about the SDM tool?
- What sort of role have you played in its development and implementation so far?
- What have been the challenges you've faced? (have these been overcome and if so, how?)
- Can you tell me about your experience of using the tool?
    - Prompts - how do you use it with patients?
    - Do you let the patient guide the use or do you take the lead when using it?
- Do you feel the tool has influenced your practice at all?
    - Prompts- In what way?
    - Has it made any difference to the amount of time spent in consultation?
    - Has it made any difference to your shared decision-making practice?
    - Do you feel it has made any difference to the patients?
- Were there any challenges when using the tool?
- How did you find recording the outcomes and use of the tool on EMIS? (any problems?)
- Do you think anything about the tool can be improved?
    - Prompt- need for training

Conclusions

- Is there anything else you would like to talk about that you think might be important or relevant to SDM for OA?

Thank you and check ok to finish.
