# Supplementary material for: Adaptation and Implementation of a Shared Decision-Making Tool From One Health Context to Another: Partnership Approach Using Mixed Methods
Source: J Med Internet Res. 2023 Jul 5;25:e42551. doi: 10.2196/42551 (PMC10357316; doi:10.2196/42551)
Supplement: Multimedia Appendix 4 [file jmir_v25i1e42551_app4.docx]

This is a Multimedia Appendix to a full manuscript published in the J Med Internet Res. For full copyright and citation information see <http://dx.doi.org/10.2196/42551>

*Summary of themes with mapping to the Theoretical Domain Framework V 2.0*

| **Overarching Themes** | **Subtheme** | **TDF domain mapped to** | **Illustrative quotes** |
| --- | --- | --- | --- |
| **Barriers** | | | |
| Barriers to the development and adaptation of the SDM tool | Conflicting priorities and perspectives of the developers of the original tool and the team adapting the tool for the local area | - Environmental context and resources - Goal priority - Knowledge - Memory attention and decision - Optimism - Intentions | *‘We thought we could just do a quick adaptation of the [original tool]’ (Interview B)*    *‘…the innovator, er, with the idea, (…) has struggled really with us making [the tools] our own and not really involving [them] enough in that process. (…) when it came to then that final bit of saying, right (…), we’re going to start using this (…) [the innovator] then said, no I’m not happy for you to launch and, er, and that’s taken months, months and months.’ (Interview E)*    *‘…one of the massive lessons learned for me is how complicated and difficult stuff is around intellectual property. (…) I think we [cross organisation collaborators] were all just sort of you know, oh let’s just work together, let’s just you know this will be fine you know. None of us really have much of a clue about IP and so you know and actually if had all worked well nobody would have been bothered.’ (Interview E)* |
|  | Perceptions of pressure to complete and release the SDM tool due to time and budget restrictions in the CCG | - Environmental context and resources - Goals - Memory attention and decision | *‘…. we’ve got 80% of a really good thing, and then we’ve gone ‘right now we’ve got to do the hip, and now we’ve got to do the shoulder, because we’ve only got this much time and the money’s running out, and we’ve got to do this by a certain date (...) I felt like a little bit of the leadership stuff was really always desperate to get it finished and get it out there’ (Interview G)*    *‘…when we got this tool, it was so bad in terms of the content, in terms of the structure and in terms of everything that we should have just started again. We should have just wiped it and said, ‘This is the idea but let’s come up with a whole new graphic and a whole new thing.’ Unfortunately, when I joined that, this was the view. ‘We’ve got a licence to use this tool. We’re going to develop it.’’ (Interview H)* |
|  | Conflicting time demands and clinical perspectives of the collaborators in the CCG | - Environmental context and resources - Social or professional role and identity | *‘Historically it is a very difficult group of people to bring together, because you’re bringing people from very different belief systems’ (Interview G).* |
|  | Being limited by how many technical and visual adaptations could be made to the original tool | - Environmental context and resources | *‘…the technical side of things... there were a lot of things I kept on saying to change and then Y would come back and say, ‘Well, the people that are developing the website can’t do this.’ You’re like, ‘The font doesn’t even fit in that section.’ (…) I think that was because we were a bit tethered to [the original tool].’ (Interview H)* |
| The barriers to implementation | SDM not a defined and resourced ambition for the NHS | - Environmental context and resources - Goal priority | *‘…the limitations for me are it’s [shared decision making] not been a properly defined ambition of our system that we will do our upmost to embed the principles of practice of shared decision-making in any environment where it may bring value. You know, we haven’t committed to that. So we’re trying to do it a little bit on the side, we’re trying to use the leave as we can do, we’re trying to use the resources that we have(…) It’s not a core thing.( Interview D)*    *‘…. it [the tool] might work really, really well if it’s digital, and it’s embedded, and someone takes responsibility for it, and it’s regularly updated. (...) but I just – I’m not sure, in the NHS, if we’re at that point where we do regularly keep things updated’ (Interview A)*    *‘... I’m hoping that that work [MSK project] will continue. But I do worry that if there isn’t somebody who’s doing a lot of that project management stuff, it just all grinds to a halt. (...) it depends on what happens really, and also the CCG’s being abolished and there’s always change…’ (Interview E)* |
|  | People in leadership positions were not bought into the SDM tool | - Environmental context and resources - Social or professional role and identity - Beliefs about consequences - Optimism | *‘I don’t have pride in what we’ve built. I think this would be reputationally negative for me if I tell everyone about it (Interview G)*    *‘... not quite understanding where it’s [original tool] come from and what the purpose is, is a little bit challenging’ (Interview A)* |
| Barriers to the use of the tool for clinicians | Limited awareness of the tool amongst clinicians | - Environmental context and resources - Social or professional role and identity - Knowledge | *‘…well [programme manager] just emailed it to people and said, ‘can you spread this word?’, rather than there was a big like press release thing, which was talked about but never quite happened. Then the thing was that version that [they] sent out on the day […], two of the links on the main home page didn’t work they sent you to a ‘this page doesn’t exist’ (Interview G)* |
|  | Challenges with the web address and finding the online tool by web search | - Environmental context and resources - Knowledge | *‘…with the launch and with the website, you can’t Google it... if you go on the CCG website and you go on search, it doesn’t even come up on there. You’re relying on this particular [SDM tool] and it’s not necessarily the most intuitive website address’ (Interview H)*    *‘I had to look each time on my email for the link was time consuming specially [sic] work across 2 different jobs.’ (Survey 20)* |
|  | Usability issues- challenges navigating the online and paper versions | Does not map to TDF domain | *‘[it] can be challenging navigating the website’ (Survey 8)*    *‘Difficult to guide patient through document fully as quite large’ (Survey 7)*    *‘The paper one is massive to print and post, and not that easy to use’ (Survey 4)*    *‘...I think we pay lip service a lot to accessibility and actually loads and loads and loads of things come out from all sorts of places and you know, I look at them and I think oh well, that’s no good for my practice (...) [the paper version] It’s just way too long. It’s a whole, it’s a novel, isn’t it? If you were to print it all out, it’s like that thick…’ (Interview C)* |
|  | Perception the SDM tool was not as user-friendly as other SDM tools | - Intentions - Beliefs about consequences | *‘when I was trying to make myself use it and send it to some people because I want to, like I’ve been involved in it, I would send ours, but in general I would send [another national SDM tool] because I think it’s just a little bit more user-friendly. I feel like we got kind of like 80% towards like a really, really good thing, […] but we just didn’t quite get it simple enough, and we didn’t quite get it kind of user-friendly enough.’ (Interview G)* |
|  | Limited time to discuss tool in consultations | - Environmental context and resources | *‘I’m signposting to the tool (…) showing them how to use it and then I’m sending them away. (…) Rather than spending that time trying to explain all those things to a patient. So it’s not more time; it’s just a different use of my time.’ (Interview F)*    *‘Needs follow up sessions to discuss. Difficult to guide patient through document fully as quite large’ (Survey 6)* |
|  | Clinicians having multiple tools to remember | - Memory attention and decision | *‘...certainly as GPs, we are rammed with so much stuff from everywhere and then it keeps changing. It’s just an absolute nightmare’ (Interview B)*  *‘I think they [patients] just get tired with the number of like links to click and things to kind of go on’ (Interview G)* |
|  | Negative perceptions of SDM tools generally | - Environmental context and resources - Social or professional role and identity - Beliefs about consequences | *‘… they [surgeons] felt like it [shared-decision making], at its worst, it was restricting patients from surgery because they haven’t done this, this, this and this (…) I think shared decision-making has got a bit of a toxic thing from the surgeons actually’ (Interview H)* |
| Perceived barriers to the use of the tool for patients | Issues with accessibility of current tool | - Environmental context and resources - Goal priority | *‘...even though I was involved in the development, I think it’s really not really that easy to use for my demographic cos I have a multi-cultural deprived population so, it’s reams and reams of reading (...) I think it’s, I do think it’s too long erm I do think that reading is a problem.’ (Interview C)*    *‘It is probably predominance of elderly people that are not comfortable with accessing things online, but you also get some generally not younger people, but more middle-aged people who just prefer not to access things online. They want it on a piece of paper and I think that’s tricky because to get that amount of information on a piece of paper is really tricky’ (Interview F)*  *‘Some patients have struggled to access the link and some patients struggle to interpret the tool (when sent the link in a text message over the phone). I think it works better to discuss through face to face but this isn't always an option.’ (Survey 13)* |
|  | Need for mobile optimisation | - Environmental context and resources - Beliefs about capabilities | *‘The information more accessible to read on a phone screen (rather than webpage) [because] often it is texted to patients’ (Survey 13)*    *‘the patients who said they’ve accessed it on their phone, it’s tricky because there’s so much information (...) So what I tend to do now is either write down the link and they’ll take it home and stick it in a web browser on their laptop, on their iPad, whatever it is, or I’ll use accuRx and I’ll send it (…) because there’s so much detail and so many bits to click on, I think it’s quite hard to access on a phone (...) I just make sure that I send it. I give them the link, or I send it to their email so they can open it up fully, rather than just trying to scroll on their phone, which they find difficult.’ (Interview F)* |
|  | Perception it is not appropriate to use the SDM tool with patient group | - Intention | *‘With the patient population I work with (students) I have had no call to use it as yet’ (Survey 6)* |
|  | Perceptions of Socio-cultural influences on patient engagement with SDM | - Social influence | *‘So there’s patients that want or are used to an old school sort of didactic medicine, an old probably white male posh doctor looking over his glasses telling you what you have to do. Some of that might be cultural as well, I do find that in certain cultures there is more of an expectation that the doctor is God kind of thing and they’ll just tell you what to do (...) I don’t think it’s a massive barrier. With time I think you can talk to those people and help them understand what the options are, and that might be less to do with the shared decision making but more to do with an expectation of a bio-medical realm of medicine where there’s always a fix, if it’s broken there’s a solution rather than, you’re just 60 years older than you were before and stuff is painful.’ (Interview G)* |
| **Current facilitators and factors suggested to improve future uptake and implementation** | | | |
| Facilitators to development and adaptation of the SDM tool | Skills of collaborators | - Environmental context and resources - Social or professional role and identity - Skills | *‘… [ researcher] got involved which was brilliant and started to bring in the proper research. And so we’ve made the product much, much, much better and based on facts and evidence really’ (Interview B)* |
|  | Group identity | - Social or professional role and identity | *‘...what I have done is asked [innovator] to join the shared decision making steering group, (...) I think we’ve got to get [innovator] really close, we’ve got to work with [them] (...)I think if we can get a good relationship with [innovator] (...) then [they] might then think, oh well yeah okay let’s let [other CCG] use it and let’s do one with neurology.’ (Interview E)* |
|  | Accessing funds to adapt the SDM tool and make it more accessible | - Environmental context and resources | *‘... shared decision-making tools are really wordy, inevitably there’s loads of information you know. So what we’ve done I got some funding to produce some videos, er, so that for people who don’t like reading lots of wordy things but would maybe be happier watching a video, we though that that would be a good approach. And, er, also [GP collaborator] was really keen that we translated these videos so they were available in lots of different languages. So that we weren’t excluding people whose first language wasn’t English…’ (Interview E)*  *‘I got some, managed to secure some funding so that I could pay (...) to release some of [Researcher's] time to do all the evidence side of it, to make sure that the actual tools are really evidence based.’ (Interview E)* |
| Facilitators to implementation of the SDM tool | Organisational culture supports SDM | - Social or professional role and identity | *‘ .... it is interesting because obviously X is a Trust, it’s a hospital Trust is really into, one into innovation, and two is into being a kind of commercial business type organisation. Er, and so they are encouraging their staff to come up with ideas and then they’re really keen to market and to promote those ideas and bring money into spread that good practice.’ (Interview E)*    *‘...so I’m trying to create the culture of shared decision-making within which we could have our [SDM tools] as a real exemplar of how to do it and these are the benefits, because it really helps when pitching particularly to medical people. Erm if you can clearly demonstrate the evidence that they’re making an impact, a positive impact. Erm so yeah, so trying to create the environment or the [SDM tool] to grow.’ (Interview D)* |
|  | Access to resources to support SDM in the local area which supported the commissioning of the tool | - Environmental context and resources - Social or professional role and identity - Skills | *‘…we put in a bid and we were accepted to be an accelerator site. Er, as part of NHS England and improvements so we became shared decision-making accelerator and as part of that we had, er, two one-day conference, well, training events. And then we had a number of virtual training events and for that work I established a steering group. (...) and we also put a bid in and was accepted for a mentor, shared decision-making mentor (...) it was a national scheme and you know we were lucky to be part of it. And there is a toolkit which the NHS England and improvement produced and I used that toolkit, er, to plan the shared decision making, the MSK shared decision-making work. (...) It’s about having a prepared public, so the public are, er, ready to be in much more of a joint decision making, er, relationship with the clinician. Er, and it’s then also about providing, er, tools to help support so that people understand, both the clinician and the patient have a good understanding of what the evidence says around the benefits, risks, er, of different treatment options…’ (Interview E)* |
|  | Demonstrating evidence that the tool is well received and can improve patient outcomes and resource use in the local area | - Beliefs about consequences - Reinforcement | *‘I was hoping (...) that the results from this study would demonstrate that patients really liked it and found it really useful and then my follow-up emails to all the consultants would be – ‘Look guys, it’s been really useful from the physios or the GPs and patients like it.’’ (Interview H)*    *‘... if we can show that they [shared decision making tools] improve the satisfaction of patient outcomes and they reduce some activity and therefore save some money and the surgeons feel happier, that they’re really more confident, that they’re operating on people that really wanna be operated on which means they’ll be less likely to have dissatisfied customers at the end of it, if it ticks all of those boxes then we can make a case to why it needs someone who needs to keep an eye on it every now and again and update the links or whatever. So the sustainability all threads from evidence of benefit, evidence of value.’ (Interview D)*    *‘... if your research shows that for some people and some clinicians it’s helpful then I think [the innovator] will feel you know pleased by that and will be thrilled that you know [their] baby, er, is being used by patients and it is helping patients and helping clinicians..’ (Interview E)* |
|  | Clinicians and people in leadership positions and clinicians believe SDM can improve patient outcomes and reduce resource use | - Memory attention and decision - Skills - Optimism - Beliefs about consequences - Environmental context and resources - Knowledge | *‘...trying to do a decent SDM without enough information is actually really difficult’ (Interview B)*    *‘….patients remember about 50% of what we tell them and 50% of what they remember is wrong, (...) it shows us even more why a), the shared decision making is important because if we’re not doing shared decision making the patients are going to understand less than that, but b), talks like this is really important because I believe if you can send them away with something that backs up what you talked about then it gives them another little bit of stuff to take onboard. ’ (Interview G)*    *‘…for me shared decision-making is an absolutely crucial way that we can help erm people have better outcomes and reduce demand on our system.’ (Interview D)*    *‘I think that by involving patients much more in their decisions about their care, and by providing them with the information about them with the information about the pros and cons, the risks and benefits they’ll be making much more informed choices and I think that will lead to much higher value, er, care. Because they won’t choose things that aren’t going to work for them.’ (Interview E)* |
|  | People in leadership positions motivated to increase awareness and engagement with the SDM tool | - Social or professional role and identity - Knowledge | *‘I know I’m using it and I know that I’ve cascaded that information, but it’s probably something that needs to be brought up in people’s observed practice and their one-to-ones and their PDR and things like that to make sure that it is being embedded.’ (Interview F)* |
|  | Suggested improvements to where in the care pathway the SDM tool is delivered | - Goal priority - Knowledge | *‘Try and push for it to be used more in primary care’ (Survey 12)*    *‘… I think the challenge in FCP [First Contact Physiotherapy] is that I send the [SDM tool] after diagnosis, usually at the consultation where we are discussing what they would like to do next in terms of management. I think it would be more useful if the patient had read the tool prior to attending this appointment. But hopefully it will be useful for them when reconsidering their options further down the pathway.’ (Survey 2)* |
| Facilitators to use of the SDM tool for clinicians | Clinician had positive perspectives and experiences of using the SDM tool | - Knowledge - Beliefs about consequences - Emotion - Goals | *‘Gives a clear idea where the [patient] is to inform shared decision making’ (Survey 1)*    *‘I think it's a really good tool to help empower our patients to make their own decisions and take control of their treatment.’ (Survey 11)*    *‘I think this a vital part of our consultation and very useful for patients’ (Survey 20)*    *‘I am happy with it and find it very simple to navigate. The colourful nature and diagrammatic forms help patients to engage as well’ (Survey 15)*    *‘easy to navigate pages. Clear and concise material content.’ (Survey 15)* |
|  | Making improvements to the content and usability of the SDM tool | Not mapped to the TDF | *‘It could be clearer regarding mild/moderate/severe categories e.g what constitutes mild/moderate/severe disease.’ (Survey 2),*    *'The 'alternative options' could be accessed in a different link, pt's often don't read 'not available on the NHS' and then start asking for platelet injections/stem cell treatment etc.’ (Survey 11).*  *‘Make it clearer how to access CSIs and TKRs [Total Knee Replacements] if they wish to.’ (Survey 11)*    *‘smaller/printable’ (Survey 14)*    *‘hard to get the balance right - sometimes the amount of information can be overwhelming to patients but hard to know what to get rid of. As concise information as possible’ (Survey 13)*    *‘I think 1 simple sheet of paper with options for pt [patients] to consider would be more easy for pts to use’ (Survey 14)*    *‘... I’ve sat on quite a lot of patient forums and patient groups and I know patients get very confused with all sorts of fancy, weird things and just need things very simple and very easy with a purpose. (…) So erm, it’s just about making that message clear about what it is and why the patient needs to use it.’ (Interview A)* |
|  | Using existing systems (e.g., Accurx templates, Remedy website, and letters to patients) to support visibility and use of the SDM tool | - Environmental context and resources - Social or professional role and identity - Knowledge | *‘.... as GPs erm, it needs to be clearly on our [referral system] page which is where we look everything up (…) you kind of need one place where people go erm, and the thing that we’ve done in our surgery is the accuRx template and we’ve just got a direct link for hips and knees but, again, it’s about GPs being – remembering it.’ (Interview B)*    *‘the link now is on AccuRx so easier to send out. cards for patient might be useful we can give them out’ (Survey 20)* |
| Potential facilitators to use of the SDM tool for patients | Passive and active approaches to increasing awareness of the SDM tool amongst patients | - Environmental context and resources - Social or professional role and identity - Knowledge | *‘I take the lead in using it because they’re not aware of it’ (Interview F).*    *‘I didn’t expect the Orthopaedic consultants to actually use it in clinic actually. What I wanted was for it to be in every single clinic letter’ (Interview H)*    *‘I thought the whole point was that, that you have a conversation with a healthcare worker or a healthcare professional, that it’s a joint thing but yeah, if you’re going to be sat at home with reams of paper, reading through a shared decision-making tool, I’m not quite sure how that will work. (...) I think it’s pr-, - it’s probably a better tool to have digitally with someone. I think it’ll work a lot better that way than having paper versions and – yeah, and posting it to people.’ (Interview A)* |
